# Suppression of Dual Specificity Phosphatase I Expression Inhibits Hepatitis C Virus Replication

**DOI:** 10.1371/journal.pone.0119172

**Published:** 2015-03-23

**Authors:** Jung Eun Choi, Jung Hyun Kwon, Jung-Hee Kim, Wonhee Hur, Pil Soo Sung, Sang Wook Choi, Seung Kew Yoon

**Affiliations:** 1 The Catholic University Liver Research Center & WHO Collaborating Center of Hepatitis, The Catholic University of Korea, Seoul, Republic of Korea; 2 Department of Internal Medicine, Incheon St. Mary’s Hospital, The Catholic University of Korea, Incheon, Republic of Korea; 3 Laboratory of Immunology and Infectious Diseases, Graduate School of Medical Science and Engineering, KAIST, Daejeon, Republuc of Korea; 4 Department of Internal Medicine, St. Paul Hospital, The Catholic University of Korea, Seoul, Republic of Korea; 5 Department of Internal Medicine, Seoul St. Mary’s Hospital, The Catholic University of Korea, Seoul, Republic of Korea; Tulane University Schiool of Medicine, UNITED STATES

## Abstract

It was reported that dual specificity phosphatase 1 (DUSP1) is specifically upregulated in the liver of patients with chronic hetpatitis C virus (HCV) infection who do not respond to peginterferon (PegIFN) treatment. Here, we have investigated the role of DUSP1 in HCV replication in hepatoma cells stably expressing the full HCV replicon (FK). DUSP1 was silenced in cells harboring the FK replicon using a lentiviral vector encoding a DUSP1-specific short hairpin RNA (LV-shDUSP1). We demonstrated that knock-down of DUSP1 significantly inhibited HCV RNA and protein expression. Also, DUSP1 silencing enhanced the expression of phosphorylated signal transducer and activator of transcription 1 (phosho-STAT1) and facilitated the translocation of STAT1 into the nucleus. The mRNA expression levels of myxovirus resistance protein A (MxA), 2'-5'-oligoadenylate synthetase 1 (OAS1), ISG15 ubiquitin-like modifier (ISG15), chemokine C-X-C motif ligand 10 (CXCL10), and ubiquitin-specific protease 18 (USP18) were also accelerated by silencing of DUSP1. Furthermore, combined with the IFN treatment, DUSP1 silencing synergistically decreased the levels of HCV RNA. These results suggest that suppression of DUSP1 expression enhances phosphorylation and nuclear translocation of STAT1, resulting in increasing expression of interferon-stimulated genes (ISGs), which synergizes with IFN's antiviral effect against HCV. In conclusion, DUSP1 is involved in the antiviral host defense mechanism against a HCV infection and thus DUSP1 might be a target to treat chronic HCV infection.

## Introduction

Hepatitis C virus (HCV) is a major cause of chronic liver disease because chronic HCV infection can progress to liver cirrhosis and hepatocellular carcinoma [1]. The current standard treatment for chronic HCV infection is a combination of peginterferon (PegIFN) and ribavirin. However, approximately 50% of patients infected with HCV genotype 1 do not achieve a sustained virologic response (SVR) to combination therapy [1–3]. Recently, new direct-acting oral agents have been developed as an alternative to PegIFN for HCV infection [4–6], but the possibility of mutations conferring resistance [7] represents a challenge, as no therapy capable of eradicating infection independent of genotype has yet been established as effective [5]. Therefore, host factors contributing to HCV replication represent ideal targets, but few have yet been reported.

A polymorphism in the *IL28B* gene was reported to affect significantly responsiveness to PegIFN treatment [8,9]. In addition, differences in the expression of host-specific genes between responsive and nonresponsive patients may also identify potential therapeutic targets. In fact, several genes are upregulated in the liver tissue of patients who later do not respond to HCV treatment [10–12]. Many of these genes are interferon-stimulated genes (ISGs), whose expression levels are consistent with a link between interferon (IFN) responsiveness and treatment efficacy [10]. The expression levels of a subset of eight genes, including dual specificity phosphatase 1 (DUSP1) and ubiquitin-specific protease 18 (USP18), have previously been used to predict the treatment response of patients with chronic hepatitis C [10]. Silencing USP18 prolongs the phosphorylated state of signal transducer and activator of transcription 1 (STAT1) and enhances the expression of ISGs in response to IFN-α [13].

DUSP1 is a mitogen-activated protein kinase phosphatase (MKP) that de-phosphorylates mitogen-activated protein kinases (MAPKs), including extracellular signal regulated kinase (ERK), c-Jun N terminal kinase (JNK), and p38, with distinct substrate specificity [14]. DUSP1 is also thought to be involved in IFN response [10,15].

However, little is known about the role of DUSP1 in the liver [16,17]; in particular, the association of DUSP1 with HCV infection remains unclear. Also, the relationships between IFN and DUSP1-associated signaling have not been elucidated. In the present study, we investigated whether DUSP1 is a host factor influencing the replication of HCV using cells stably expressing the FK replicon.

## Materials and Methods

### Cell culture

The FK replicon (a gift from Sung Key Jang, Pohang University of Science and Technology, Pohang, Republic of Korea) is a full-length HCV genotype 1b sequence that replicates autonomously in human Huh7 hepatoma cells. The FK replicon and Huh7 cells were maintained in Dulbecco’s modified Eagle’s medium (DMEM; Invitrogen, Carlsbad, CA) supplemented with 10% fetal bovine serum (FBS) and 1% antibiotics (100 μg/mL penicillin and 0.25 μg/mL streptomycin) in a humidified incubator at 37°C with 5% CO_2_. FK replicon cells were selected by growth in medium containing 500 μg/mL G418 sulfate.

### Generation of stably expressing short-hairpin RNA (shRNA) DUSP1

FK replicon cells were infected with DUSP1 shRNA using the shRNA-lentiviral infection system (Sigma-Aldrich, St. Louis, MO). A negative control lentiviral particle (LV-cont) was also constructed. Cells in which DUSP1 expression was stably suppressed were established using shRNA-carrying lentivirus particles according to the method of Choi et al [18]. In brief, FK replicon cells were seeded at a density of 1 × 10^5^ cells per well and infected with 5 multiplicity of infection (MOI) lentiviral particles in the presence of 8 μg/mL hexadimethrine bromide (Sigma-Aldrich) overnight. Stably infected cells were selected for 2 weeks using complete medium with 500 μg/mL G418 sulfate (A.G. Scientific, San Diego, CA) and 10 μg/mL puromycin (Sigma-Aldrich). Suppression of DUSP1 expression in selected cells was confirmed by relative quantitative real-time polymerase chain reaction (PCR) and Western blot analysis.

### Transient transfection

To overexpress DUSP1 protein, we were provided pcDNA3.1-DUSP1 plasmid from kindly Un-Hwan Ha (Korea University, Chungnam, Republic of Korea). Established LV-shDUSP1-infected cells were transfected with pcDNA3.1 (Mock) or pcDNA3.1-DUSP1 using Fugene HD (Promega, Madison, WI) according to the manufacturer’s protocol. After 72 h, the cells were harvested and stored until further analyze.

### Relative quantitative real-time PCR (rqRT-PCR)

Total RNA was extracted using TRIzol reagent (Invitrogen) according to the manufacturer’s protocol. Complementary DNA (cDNA) was synthesized from 5 μg of total RNA using reverse transcriptase (Promega, Madison, WI) and random primers (Promega) and amplified using Lightcycler 480 Probes Master real-time PCR master mix (Roche Applied Science, Indianapolis, IN) in combination with Universal Probe Library (UPL) assays (Roche Applied Science). Assays were designed according to publicly available gene sequences (NCBI) using ProbeFinder UPL software (v.2.45) (Roche Applied Science). Each 20 μL PCR reaction comprised 0.4 μM target primers, 0.4 μM target UPL, 0.4 μM reference primers, 0.4 μM reference probe, and Roche real-time PCR master mix. The cycling conditions were as follows: preincubation at 95°C for 10 min, followed by 45 cycles at 95°C for 10 s, 55°C for 45 s, and 72°C for 1 s. Human β-actin and human glucose 6-phosphate dehydrogenase (G6PD) were used as reference genes. All fluorescence data were analyzed using LightCycler 4.0 software (Roche Applied Science), and C_t_ results were exported to Excel (Microsoft, Redmond, WA). Gene expression was quantified and normalized using the comparative C_t_ method.

### Western blot analysis

Cells were washed with phosphate-buffered saline (PBS) and lysed in Pro-prep (iNtRon Biotechnology, Houston, TX) containing protease inhibitors for 20 min on ice. Samples were then centrifuged at 13,000 rpm for 10 min at 4°C and the supernatant was transferred to a new tube. Protein concentration was determined by Bradford assay (Bio-Rad Laboratories, Hercules, CA). Extracted protein (30 μg) was subjected to 10% sodium dodecyl sulfate–polyacrylamide gel electrophoresis (SDS-PAGE) and transferred to nitrocellulose membranes (Whatman, Maidstone, Kent, UK). Membranes were blocked with PBS containing 5% skim milk and incubated with anti-DUSP1 (Santa Cruz Biotechnology, Santa Cruz, CA), anti-NS5A (Meridian Life Sciences, Saco, ME), anti-NS5B (Santa Cruz Biotechnology), or anti-β-actin (Sigma-Aldrich) at 4°C overnight. Each membrane was washed three times with TBS containing 0.05% Tween 20 and then incubated with 1:5000 dilution of horseradish peroxidase (HRP)-conjugated anti-mouse or anti-rabbit immunoglobin G (IgG; Santa Cruz Biotechnology). Finally, membranes were washed three times with TBS containing 0.05% Tween 20, and protein bands were visualized using an enhanced chemiluminescence system (Amersham Biosciences, Piscataway, NJ) according to the manufacturer’s instructions.

### HCVcc infection and siRNA transfection

Huh7 cells were seeded at a density of 1 × 10^6^ cells per 100-mm dish and transiently transfected with 30 nM DUSP1 siRNA (Santa Cruz Biotechnology) or scrambled siRNA (Sigma-Aldrich) by simultaneous seeding with G-fectin (Genolution Pharmaceuticals, Seoul, Republic of Korea). After 1 day of transfection, cells were infected using HCVcc for 3 days at 37°C. DUSP1 and HCV RNA expression was measured by rqRT-PCR. Also, DUSP1 protein was measured by Western blot as described above.

### Quantitiation of HCV RNA

HCV RNA was quantified using cDNA synthesized as described above, according to a previously reported method [19].

### Cell-based enzyme-linked immunosorbent assay (ELISA)

STAT1 activity was detected using a human phospho-STAT1 (Y701) immunoassay kit (R&D Systems, Minneapolis, MN) according to the manufacturer’s instructions. In brief, cells expressing LV-cont or LV-shDUSP1 were seeded into black 96-well plates at a density of 1 × 10^4^ cells per well, fixed with 4% paraformaldehyde (Sigma-Aldrich) for 20 min at room temperature, and washed three times with wash buffer. Endogenous peroxidases were quenched with 3% H_2_O_2_ for 1 h at room temperature and cells were washed a further three times with wash buffer, incubated in blocking buffer for 1 h at room temperature, and incubated with primary antibody (1:100 dilution of anti-phospho-STAT1 and 1:100 dilution of anti-STAT1) overnight at 4°C. Cells were washed three times with wash buffer, incubated with secondary antibody (1:100 dilution of HRP-conjugated antibody and 1:100 dilution of alkaline phosphatase-conjugated antibody) for 2 h at room temperature, washed a further three times with wash buffer, and developed using substrate F1 and substrate F2 for 1 h at room temperature. The plate was protected from direct light throughout the procedure. After development, fluorescence was measured at 540 nm excitation and 600 nm emission, and then at 360 nm excitation and 450 nm emission.

### Immunocytochemistry

To assess the effect of DUSP1 silencing on translocalization of STAT1, cells expressing LV-cont or LV-shDUSP1 were seeded at a density of 2 × 10^5^ cells per plate. After 1 day of seeding, cells were fixed with 4% paraformaldehyde for 30 min, washed three times with PBS for 5 min, and incubated at 4°C overnight with a 1:50 dilution of anti-STAT1 (Cell Signaling Technology, Beverly, MA) in blocking solution (1% bovine serum albumin). Cells were washed three times with PBS, then incubated with Alexa 594-conjugated anti-rabbit IgG (Molecular Probes, Eugene, OR) for 1 h at room temperature. Nuclei were detected by staining with 1 μg/mL 4,6-diamidino-2-phenylindole (DAPI; Sigma-Aldrich) for 5 min. After three washes with PBS, preparations were mounted with Kaiser’s glycerin gelatin (Merck, Darmstadt, Germany) and examined using an Axiovert fluorescence microscope (Carl Zeiss, Göttingen, Germany).

### IFN treatment

To determine whether IFN treatment affects HCV replication in the context of DUSP1 suppression, LV-shDUSP1 were seeded at a density of 1 × 10^6^ cells per 100-mm dish, cultured overnight, washed three times with PBS, then treated with fresh media containing 0, 10^2^, 10^3^, or 10^4^ IU/mL IFN-α (Sigma-Aldrich) for 3 days. Total RNA was extracted and stored at −80°C; HCV RNA was quantified by rqRT-PCR as described above.

### Statistical analysis

All experiments were performed at least in triplicate. Data are expressed as means ± SD. Statistical comparisons were made using Student’s *t*-test or ANOVA using SPSS version 14.0 software (SPSS Inc., Chicago, IL). *P* < 0.05 was considered significant.

## Results

### DUSP1 suppression inhibits HCV replication and translation without IFN treatment

**Fig 1:**
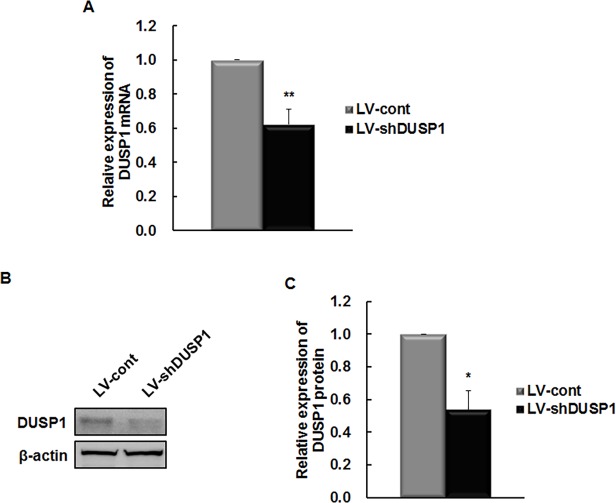
DUSP1-specific shRNA downregulates DUSP1 expression in FK replicon. To establish a cell line supporting stable HCV replication in which DUSP1 expression was suppressed, the FK replicon (full HCV genome) were infected with negative control (LV-cont) or DUSP1-specific shRNA (LV-shDUSP1). DUSP1 mRNA and protein were measured by rqRT-PCR (A) and Western blot analysis (B and C) and normalized to G6PD and β-actin, respectively. All data are representative of at least three independent experiments. *P < 0.05 and **P < 0.01 compared to the control.

To assess the effect of DUSP1 silencing on the HCV life cycle, the FK replicon was infected with LV-shDUSP1 particles. LV-shDUSP1–1, LV-shDUSP1–2, and LV-shDUSP1–3 were designed to target three different regions of the DUSP1 gene, starting at nucleotides 502, 958, and 704, respectively. Colonies of LV-shDUSP1–2 infected cells could not be obtained by puromycin selection, and LV-shDUSP1–1 did not affect mRNA or protein levels of DUSP1 (data not shown), whereas LV-shDUSP1–3 effectively downregulated DUSP1. Therefore, LV-shDUSP1–3 was used in all further experiments. LV-shDUSP1-infected cells expressed significantly lower levels of DUSP1 mRNA and protein than LV-cont-infected cells (*P* < 0.05; Fig. 1).

**Fig 2:**
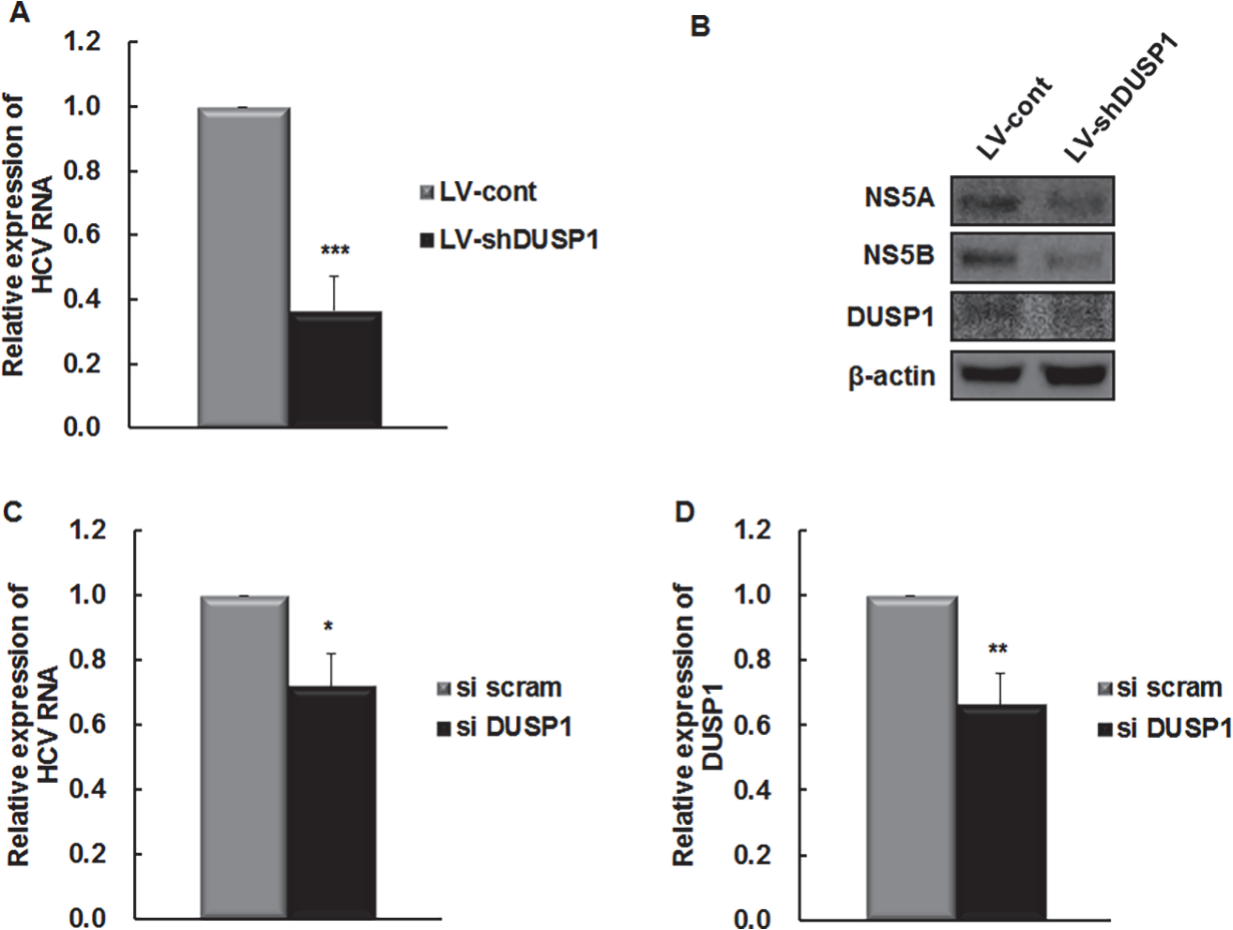
DUSP1 silencing has an antiviral effect in the FK replicon and HCV cc system. (A) HCV RNA was measured in LV-shDUSP1-infected cells by rq-RT-PCR and normalized to β-actin and expressed relative to levels in LV-cont-infected cells. (B) HCV NS5A, HCV NS5B, DUSP1, and β-actin protein were measured by Western blot analysis and normalized to β-actin. (C and D) Direct effects of DUSP1 knockdown were confirmed by transfection of DUSP1-siRNA in HCVcc system. At 72 h post-transfection, HCV (C) and DUSP1 mRNA (D) were measured by rqRT-PCR and normalized to β-actin and G6PD, respectively, then to expression in control cells. All data are representative of at least three independent experiments. *P < 0.05; **P < 0.01; ***P < 0.001 compared to the control. si scram, scrambled siRNA; si DUSP1, DUSP1 siRNA.

Next, we determined whether silencing of DUSP1 affects HCV replication and translation. HCV RNA titers were reduced by about 60% in LV-shDUSP1-infected cells relative to LV-cont-infected cells (*P* < 0.001; Fig. 2A). In addition, the expression levels of HCV proteins NS5A and NS5B were lower in LV-shDUSP1-infected cells (Fig. 2B). To confirm off-target effect of DUSP1-shRNA, DUSP1-siRNA targeting a distinct DUSP1 sequence was transfected into Huh7 cells. Subsequently, siRNA transfected Huh7 cells were infected with HCVcc and incubated for 3 days. As expected, DUSP1 siRNA transfection decreased HCV RNA levels significantly relative to scrambled siRNA-transfected cells (*P* < 0.05; Fig. 2C and Fig. 2D). Collectively, these results show that silencing of DUSP1 suppresses expression of HCV RNA and protein, identifying DUSP1 as a novel antiviral target in HCV infection even in the absence of IFN treatment.

### Downregulation of DUSP1 enhances the activity of STAT1

**Fig 3:**
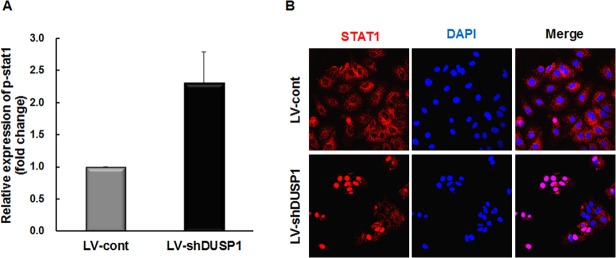
DUSP1 suppression increases STAT1 activity and nuclear translocation. Phosphorylation (A) and nuclear translocation (B) of STAT1 were assessed by cell-based ELISA and confocal microscopy, respectively. (A) Expression of phospho-STAT1 was normalized to that of STAT1 and then to the ratio in LV-cont-infected cells. (B) Red, anti-STAT1; blue, DAPI. Merged image allows assessment of nuclear localization of STAT1; original magnification ×200. All data are representative of at least three independent experiments.

To determine how DUSP1 silencing suppresses HCV expression, we examined its effects on phosphorylation and localization of STAT1 given its upregulation by IFN signaling [20]. As inhibition of nuclear phosphatases prolongs phosphorylation of STAT1 in hepatocytes [21], we hypothesized that DUSP1 suppression likely regulates STAT1 activity. Western blot analysis revealed that LV-shDUSP1 infection enhances phosphorylation of STAT1 compared to LV-cont-infected cells (*P* = 0.06; Fig. 3A). Moreover, LV-shDUSP1 infection enhances translocation of STAT1 protein from the cytoplasm into the nucleus relative to in LV-cont-infected cells (Fig. 3B). These results demonstrate that downregulation of DUSP1 enhances the activity and nuclear translocation of STAT1.

### Silencing of DUSP1 enhances the expression of ISGs

**Fig 4:**
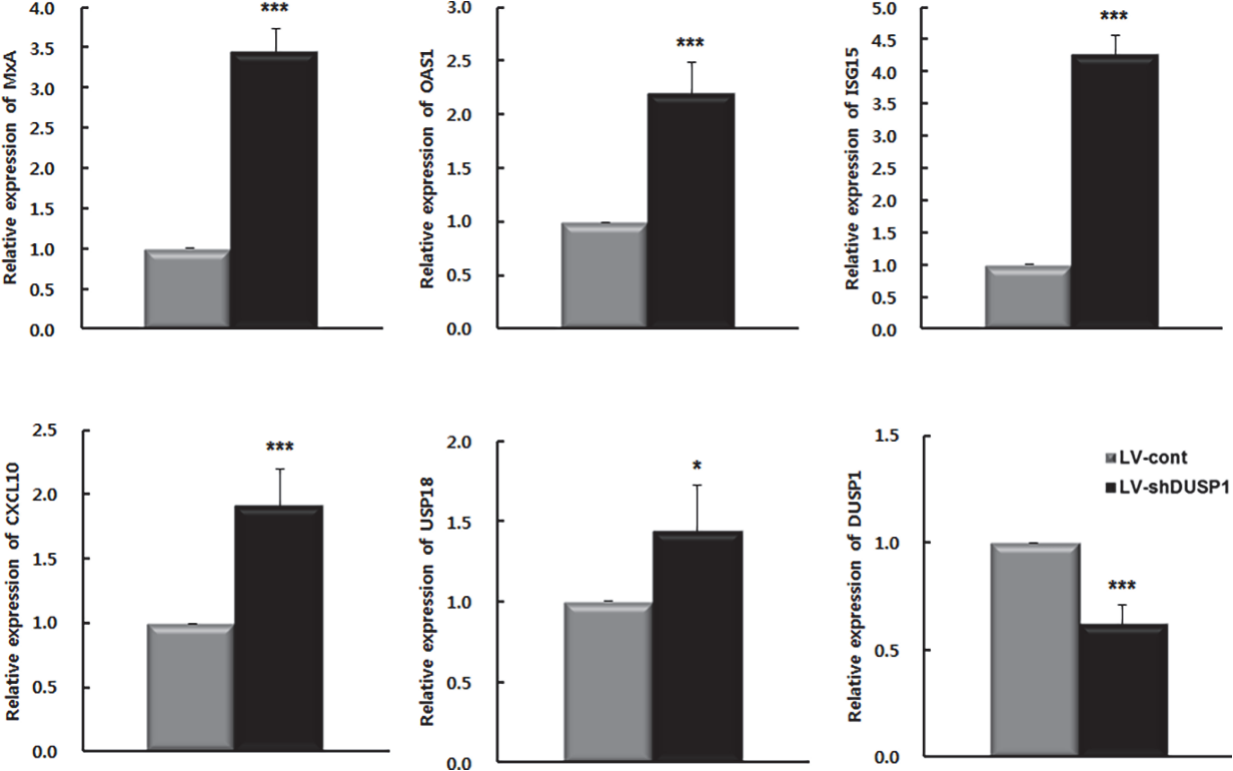
Downregulation of DUSP1 enhances ISG expression. mRNA expression of MxA, OAS1, ISG15, CXCL10, USP18 and DUSP1 were determined by rqRT-PCR and normalized to β-actin or G6PD and then to expression in LV-cont-infected cells. All data are representative of at least three independent experiments. *P < 0.05, ***P < 0.001.

Phosphorylated STAT1 heterodimers associate with interferon-regulatory factor 9 to form ISG factor 3, which translocates to the nucleus to induce ISG expression by binding interferon-stimulated response elements (ISREs) [22]. Induction of ISGs increases the antiviral effect of IFN-α [22]. Thus, we evaluated the effect of DUSP1 silencing on the transcription of ISGs. LV-shDUSP1 infection enhanced mRNA expression of myxovirus resistance protein A (MxA), 2'-5'-oligoadenylate synthetase 1 (OAS1), ISG15 ubiquitin-like modifier (ISG15), chemokine C-X-C motif ligand 10 (CXCL10), and USP18 relative to those in LV-cont-infected cells (*P* < 0.05; Fig. 4). These results suggest that silencing of DUSP1 enhances ISG expression via STAT1activation.

### Synergic effect of IFN treatment and DUSP1 silencing on HCV replication

**Fig 5:**
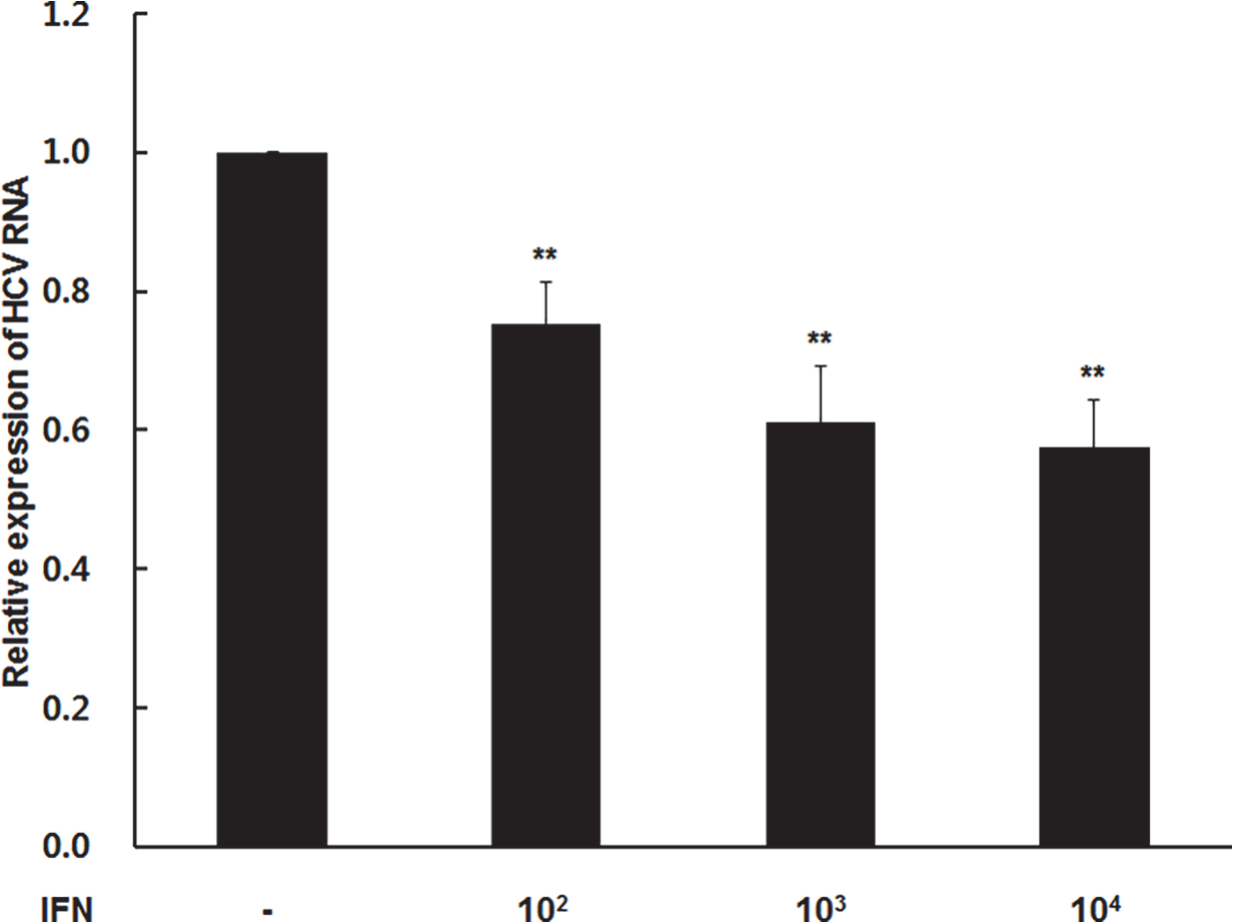
Synergistic effects of IFN-α with DUSP1 depletion against HCV. Cells were treated with 0–10^4^ IU/mL IFN-α for 72 h. HCV RNA was measured by rqRT-PCR and normalized to β-actin and then to LV-shDUSP1-infected cells not treated with IFN. All data are representative of at least three independent experiments. **P < 0.01.

The above results show that DUSP1 suppression has an antiviral effect on HCV independent of IFN treatment, but it could also enhance the expression of IFN. We therefore examined whether silencing of DUSP1 synergistically enhances the antiviral activity of IFN against HCV. LV-shDUSP1-infected cells or LV-cont-infected cells were treated with 0, 10^2^, 10^3^, and 10^4^ IU/mL IFN-α for 72 h. After treatment, IFN significantly decreased HCV RNA expression in a dose-dependent manner (*P* < 0.05; Fig. 5). These results suggest that silencing of DUSP1 has a synergistic antiviral effect on HCV with IFN treatment.

### Overexpression of DUSP1 rescue the influence of HCV replication by DUSP1 silencing

**Fig 6:**
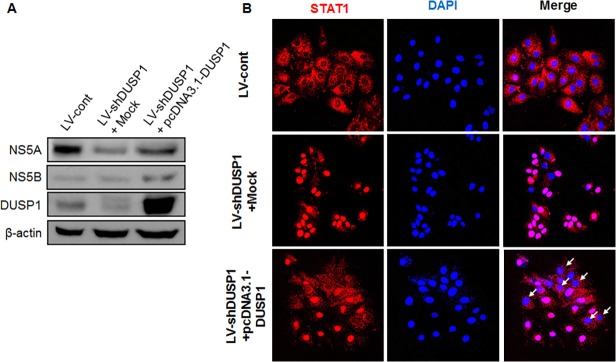
Overexpression of DUSP1 rescues anti-HCV effect by DUSP1 silencing. LV-shDUSP1 cells were transfected with pcDNA3.1-DUSP1 plasmid. (A) At 72 h post-transfection, the level of HCV proteins were determined by western blot analysis. (B) Localization of STAT1 was detected using immunocytochemistry. Red, anti-STAT1; blue, DAPI. White narrows indicate cytoplasm location of STAT1; original magnification ×200. All data are representative of at least three independent experiments.

To confirm the influence on the replication of HCV by DUSP1 silencing, we transfected with pcDNA3.1-DUSP1 in LV-shDUSP1-infected cells. After transfection, low-expression of HCV proteins was rescued by DUSP1 overexpression (Fig. 6A). In addition, accumulated STAT1 in nucleus also was dramatically released to cytoplasm by restored DUSP1 expression (Fig. 6B). Taken together, we demonstrated that the HCV replication was regulated via STAT1-dependent DUSP1 expression.

## Discussion

This study demonstrates that DUSP1 silencing inhibits HCV replication in cells expressing the HCV genome. DUSP1 silencing enhanced phosphorylation and nuclear translocation of STAT1, resulting in increasing expression of ISGs leading to suppress HCV replication. Moreover, DUSP1 silencing enhanced the antiviral effect of IFN in FK replicon cells treated with IFN.

Recently, new direct-acting oral agents for HCV have increased rates of SVR compared to prior IFN-based therapy [4–6]. However, no therapy capable of fully eradicating HCV independent of genotype has yet been introduced.

Host-targeting agents (HTAs) represent a promising new direction in the development of HCV antivirals because of their high genetic barrier to resistance and pan-genotypic antiviral activity. Also, their complementary mechanism of action suggests that HTAs may inhibit HCV in a synergistic manner with IFN and/or direct-acting oral agents. One recently developed anti-HCV HTA is miravirsen, an inhibitor of microRNA-122, which prolongs reductions in HCV RNA levels without evidence of resistance [23,24]. Similarly, NIM811, a non-immunosuppressive cyclophilin inhibitor, normalizes levels of liver transaminases and is expected to enhance antiviral activity in combination with pegIFN [25]. However, most HTAs currently in clinical trials only limit the HCV life cycle and do not eradicate infection.

Candidate targets for anti-HCV HTAs include genes whose polymorphisms or expression affect the response to IFN treatment. Within the former category, *IL28B* polymorphisms have been reported to significantly affect responsiveness to IFN treatment [8,9]. Within the latter, DUSP1 which plays a key role in the regulation of pro-inflammatory and anti-inflammatory cytokines [26], has been suggested [10]. However, the role of DUSP1 for the antiviral activity against HCV remains unclear. In the present study, the association of DUSP1 with STAT1 in the IFN signaling pathway and its antiviral role against the HCV were defined (Fig. 2 and Fig. 3). IFN-α induces the phosphorylation of STAT1 and STAT2 [27]. Silencing of DUSP1 by shRNA not only enhanced STAT1 activation but also its translocation into the nucleus, leading to increased expression of ISGs such as MxA, OAS1, ISG15, CXCL10, and USP18 and thus enhanced antiviral activity against HCV (Fig. 3 and Fig. 4).

Like DUSP1, USP18 is also expressed to a greater degree in the liver of IFN nonresponders than responders prior to treatment [10]. Silencing of USP18 by siRNA was later shown to prolong STAT1 phosphorylation and enhance ISG expression, resulting in a synergistic antiviral effect on HCV treated with IFN [13]. In the present study, DUSP1 silencing inhibited HCV replication in both the presence and absence of IFN (Fig. 2 and Fig. 5). Knocking down DUSP1 enhanced IFN expression and acted synergistically with IFN to decrease HCV RNA expression in a dose-dependent manner. Notably, the inhibitory effect of DUSP1 silencing on HCV replication increased when cells were not treated with IFN-α, unlike USP18; however, the mechanism of this effect remains unclear. Our novel findings confirm that DUSP1 is a host factor influencing HCV infection, and like ISG15 and USP18, that its influence is associated with the IFN signaling pathway. Therefore, DUSP1 may be a target for future HTA development; such drugs may be effective either alone or in combination with IFN or direct-acting oral agents.

In conclusion, this study demonstrates that DUSP1 suppression enhances phosphorylation and nuclear translocation of STAT1, resulting in increasing expression of ISGs, which synergizes with IFN’s antiviral effect against HCV. These findings suggest that DUSP1 is involved in the antiviral host defense mechanism against a HCV infection and thus DUSP1 is a candidate target for chronic HCV infection.
